# Oxysterols Versus Cholesterol in Model Neuronal Membrane. I. The Case of 7-Ketocholesterol. The Langmuir Monolayer Study

**DOI:** 10.1007/s00232-017-9984-8

**Published:** 2017-08-31

**Authors:** Anita Wnętrzak, Katarzyna Makyła-Juzak, Anna Filiczkowska, Waldemar Kulig, Patrycja Dynarowicz-Łątka

**Affiliations:** 1 Jagiellonian University, Ingardena 3, 30-060 Krakow, Poland; 2 Institute of Physics, Jagiellonian University, Łojasiewicza 11, 30-348 Krakow, Poland; 3 Department of Physics, Tampere University of Technology, P. O. Box 692, 33101 Tampere, Finland

**Keywords:** Langmuir monolayers, 7-Ketocholesterol, Model neuronal membrane, Interactions

## Abstract

Oxysterols are products of cholesterol oxidation. They can be formed endogenously (in both enzymatic and non-enzymatic reactions) as well as exogenously (delivered with food). Recent studies clearly demonstrate cytotoxic properties of these compounds, being mainly due to their incorporation into natural lipid bilayers. This process can influence mechanical and physicochemical properties of biomembrane—mainly by modifying the interactions between its components, which may result in the disruption of proper functioning of cell membrane and could lead to its degradation. Therefore, it can be assumed that oxysterols may affect the initiation of neurodegenerative diseases, including Alzheimer’s disease. However, the mode of action of these molecules at the molecular level is not fully known. To get a better understanding of the role of oxysterols in neurodegeneration, it is of great importance to examine mutual interactions between oxysterols and neuronal membrane components. One of the most promising techniques that can be used to analyze such interactions is the Langmuir monolayer technique. In this work, we have prepared an artificial neuronal membrane modeled as multicomponent Langmuir monolayer built up with cholesterol, 1-palmitoyl-2-oleoyl-sn-glycero-3-phosphocholine (POPC), and sphingomyelin (SM). To examine whether there are any changes in the membrane properties under oxidative stress, in this paper we have investigated the impact of the representative ring-oxidized oxysterol: 7-ketocholesterol (7-KC). Our results show that replacing cholesterol with 7-KC increases the interaction between molecules in the model membrane.

**Electronic supplementary material:**

The online version of this article (doi:10.1007/s00232-017-9984-8) contains supplementary material, which is available to authorized users.

## Introduction

Neurodegenerative diseases, notably affecting an increasing number of people, are currently a very serious problem in the world. Despite different symptoms of neurodegenerative processes, all inevitably lead to nerve cell damage and—as a consequence—to motor functions’ impairment (such as in Parkinson’s disease, amyotrophic lateral sclerosis, or Huntington’s disease) and disorder of cortical functions (Alzheimer’s disease) (Borlongan et al. 1995; Coyle et al. 1983). The latter often affects elderly people and leads to dementia. The World Health Organization pays special attention to the alarming fact that even 47.5 million people in the world suffer from dementia, and this number may rise up to approx. 135.5 million (www.who.int). Regardless of the literature reports in this area, the causes of neurodegenerative diseases responsible for neuronal degeneration at the molecular level are not well understood. One of the hypotheses assumes that the direct cause of neurodegenerative diseases may be related to overproduction of oxysterols, which are the oxidation products of cholesterol (Leoni and Caccia 2011). These cholesterol derivatives contain one or more additional oxygen-containing functional groups, like hydroxyl, carbonyl, carboxyl, or epoxy (Brown and Jessup 2009). Oxysterols have a long history of study, dating back from their initial characterization in the beginning of 1900s, and nowadays their role in natural and pathological processes is fairly well recognized (for a review see Brown and Jessup 2009). Oxysterols are present in healthy human and animal tissues, however at a very low concentration as compared to cholesterol. In physiological conditions, they play an important and beneficial role as they are involved in the regulation of cholesterol homeostasis by elimination of cholesterol excess from the body. However, in pathological processes (mainly related to neurodegenerative and age-related diseases) (Vejux and Lizard 2009), their level is significantly elevated and they are found to be damaging.

There is a wide array of oxysterols encountered in healthy human. They vary in their origin, i.e., oxysterols can be formed in vivo, as a result of non-enzymatic reaction, induced by reactive oxygen species (ROS), leading to the formation of ring-oxidized sterols, mainly at the 7-position (e.g., 7α-hydroxycholesterol; 7α-OH, 7β-hydroxycholesterol; 7β-OH, 7-ketocholesterol; 7-KC). They may also be produced during enzymatic reactions, forming side-chain oxidized sterols (24*S*-hydroxycholesterol, 24-OH; 25-hydroxycholesterol, 25-OH, and 27-hydroxycholesterol, 27-OH) formed by the action of the separate enzymes (cholesterol 24- and 27-hydroxylase, being P450 enzymes, and cholesterol 25-hydroxylase, being a non-heme iron-containing protein). However, exceptions to this rule do occur; for example, 25-OH and 7α-OH can be produced by in both enzymatic and non-enzymatic ways (Gill et al. 2008). In addition, oxysterols can also be delivered with food, particularly cholesterol-rich food (e.g., 7-KC, 7α- and 7β-OH, as well as α- and β-5,6-epoxycholesterol, abbr. α- and β-EPOX) (Tabas 2002). They are most probably generated non-enzymatically during food processing and cooking.

A great number of experimental studies and clinical observations clearly demonstrate cytotoxic properties of oxysterols through their different actions: mutagenic, carcinogenic, inhibition of DNA synthesis, and biosynthesis of cholesterol (Wielkoszyński et al 2006; Björkhem 2002). Biological activity and physicochemical properties of oxysterols are different from cholesterol and can affect cell membranes by building up into their structure, which in turn may change membrane structural properties, fluidity, and permeability (Mitomo et al. 2009; Sottero et al. 2009; Kulig et al. 2015).

**Fig. 1:**
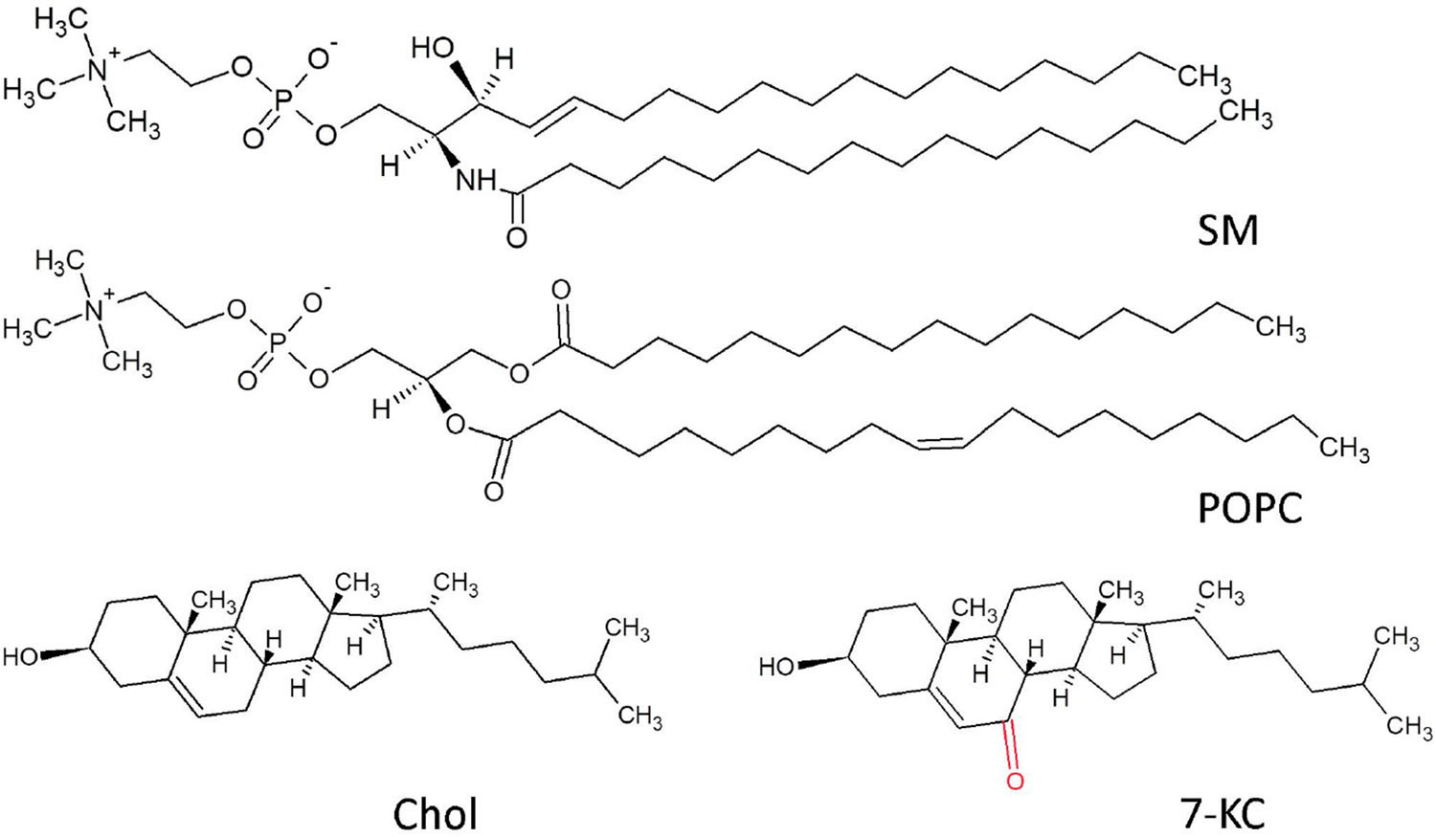
Chemical structures of sphingomyelin (SM), 1-palmitoyl-2-oleoyl-sn-glycero-3-phosphocholine (POPC), cholesterol (Chol), and 7-ketocholesterol (7-KC)

This paper is aimed at conducting extensive research on the effect of selected oxysterol on natural membrane lipids as well as on the structure and surface properties of the model membrane of neuronal cell. In order to understand better the mechanisms responsible for neuronal degeneration, it is necessary to examine many fundamental processes occurring in the nerve cells and make a comparison of mutual interactions in model biological systems. Therefore, we have modeled an artificial neuronal membrane as a 3-component system of cholesterol, 1-palmitoyl-2-oleoyl-sn-glycero-3-phosphocholine (POPC), and sphingomyelin (SM) using the Langmuir monolayer technique (Gaines 1966). POPC was chosen as the most abundant phospholipid present in mammalian cell membrane, whereas SM was selected as a typical neuronal cell lipid (Barenholz and Thompson 1999). In order to find out how oxysterols influence neuronal membrane, we have replaced cholesterol by representative ring-oxidized oxysterol (7-ketocholesterol, 7-KC) (Fig. 1) and examined the properties of such a modified membrane.

The use of the Langmuir monolayer technique (Gaines 1966) in the context of neurodegenerative diseases is not common. The application of this method in biomedical sciences is based on fabricating a monolayer (film), built from membrane lipids, which may serve as a simple, physical model of the cellular membrane (Maget-Dana 1999; Stefaniu et al. 2014; Nobre et al. 2015). Although the natural cell membrane is a bilayer and the Langmuir monolayer represents only its one leaflet, however, a strong correlation has been found between monolayers and bilayers. Due to the so-called *‘monolayer*–*bilayer correspondence’* (Marsh 1996; Brockman 1999; Feng 1999), monolayers reveal similar properties to bilayers at surface pressures of 30–35 mN/m. Therefore, under these conditions, Langmuir monolayer can successfully mimic a bilayer system. The advantage of using such a multicomponent surface film as a biomembrane model is its simplicity of preparation and possibility of a continuous, easy, and precise control of both quality of the surface and such parameters as molecular packing, physical state, lateral pressure, and composition. Therefore, this method enables simple modification of the physicochemical parameters during the experiment, which gives the possibility to obtain the conditions most similar to physiological (Gaines 1966; Maget-Dana 1999; Eeman and Deleu 2010). Thermodynamic results of the Langmuir monolayer experiments provide information on the nature and strength of interactions in the system of interest (Dynarowicz-Łątka and Kita 1999).

A plethora of successful examples of the use of model biological membranes to study the mode of actions of different biomolecules (including drugs acting on membrane level) can be found in the literature. For example, the affinity of alkylphosphocholines—synthetic antitumor lipids—to prostate cancer membrane was confirmed by Langmuir monolayer experiments (Wnętrzak et al. 2014). Another example is the antimalarial activity of cyclosporin A studied with model Langmuir membrane (Dynarowicz-Łątka et al. 2015). Changes in membrane organization were also reported for edelfosine (Hąc-Wydro and Dynarowicz-Łątka 2010a, b; Dynarowicz-Łątka and Hąc-Wydro 2014), amphotericin B (Hąc-Wydro et al. 2009; Foglia et al. 2014), mycosubtilin (Nasir 2012), and many other bioactive compounds (for a review see Nobre et al. 2015). All these examples show that artificial membrane is a very useful tool to study mutual interactions between drugs and membrane components and allows confirming and understanding the results observed in in vivo studies.

## Experimental

### Materials

#### Investigated Molecules

The following materials were purchased and used: 7-ketocholesterol (7-KC), cholesterol (Chol), sphingomyelin (SM), and 1-palmitoyl-2-oleoyl-sn-glycero-3-phosphocholine (POPC) (all from Avanti Polar Lipids). All of these compounds were of high purity (>99%) and used as received. Chol, POPC, and 7-KC were synthetic, while SM was isolated from porcine brain.

#### Solvents

Chloroform and methanol (POCh) with a purity of ≥99% were used to wash the Langmuir trough. Solutions were prepared using spectroscopic grade chloroform (purity ≥ 99.9%, HPLC dedicated) containing methanol as a stabilizer (POCh).

#### Subphase

In routine experiments, ultrapure water from demineraliser HLP (Hydrolab), with a conductivity <0.06 mS/cm, was used as a subphase. In individual measurements, a buffer was used to adjust subphase pH (Teorell and Stenhagen 1938).

### Methods

#### Langmuir Monolayer Experiments

The investigated compounds were dissolved in chloroform with a typical concentration of 0.2–0.5 mg/ml. Mixed solutions were obtained by mixing appropriate volumes of the respective stock solutions. Models of neuronal membrane were prepared by mixing SM with POPC in proportion 1:1. Langmuir monolayers were obtained by spreading an aliquot of the above-mentioned solutions with a Hamilton microsyringe onto the surface of ultrapure water. The π–A isotherms were recorded using double-barrier Langmuir–Blodgett trough (KSV NIMA, Helsinki, total area = 273 cm^2^) placed on an anti-vibration table. In routine measurements, monolayers were compressed with the barrier speed of 20 cm^2^/min. Surface pressure was measured using a Wilhelmy plate made from ashless chromatography paper (Whatman Chr1) as the surface pressure sensor, with a sensitivity of ±0.01 mN/m. Temperature of the aqueous subphase was held constant by a circulating water system from Lauda. Each measurement was repeated 2–3 times to ensure high reproducibility of the obtained isotherms to ±2 Å^2^/molecule.

Structure of monolayers was visualized with a Brewster angle microscope (BAM) using an ultraBAM instrument (Accurion GmbH, Goettingen, Germany) equipped with a 50-mW laser emitting *p*-polarized light at a wavelength of 658 nm, a 10× magnitude objective, a polarizer, an analyzer, and a CCD camera. The spatial resolution of the BAM image was 2 µm. The BAM instrument was installed over a KSV 2000 700 cm^2^ double-barrier Langmuir trough (KSV, Helsinki, Finland). The procedure was identical to that described above.

## Results

In order to characterize model membranes and analyze the interactions between constituent lipids, in the first step we looked at the behavior of particular lipids (SM, POPC, 7-KC, and cholesterol) in Langmuir monolayers. Then we increased the complexity of the system to 2-component (SM/POPC, SM/sterol, POPC/sterol) and 3-component (cholesterol/SM/POPC and 7-KC/SM/POPC) mixtures.

### Langmuir Monolayer Characteristics of Neuronal Membrane Lipids

A typical neuronal membrane consists of sphingolipids (exemplified by SM), glycerophospholipids (exemplified by POPC), and cholesterol (Jamieson and Robinson 1977).

Our intention was to model neuronal membrane, and therefore in our experiments we used sphingomyelin isolated from brain. Sphingomyelin from this source is not frequently used, contrary to that isolated from chicken egg.

**Fig. 2:**
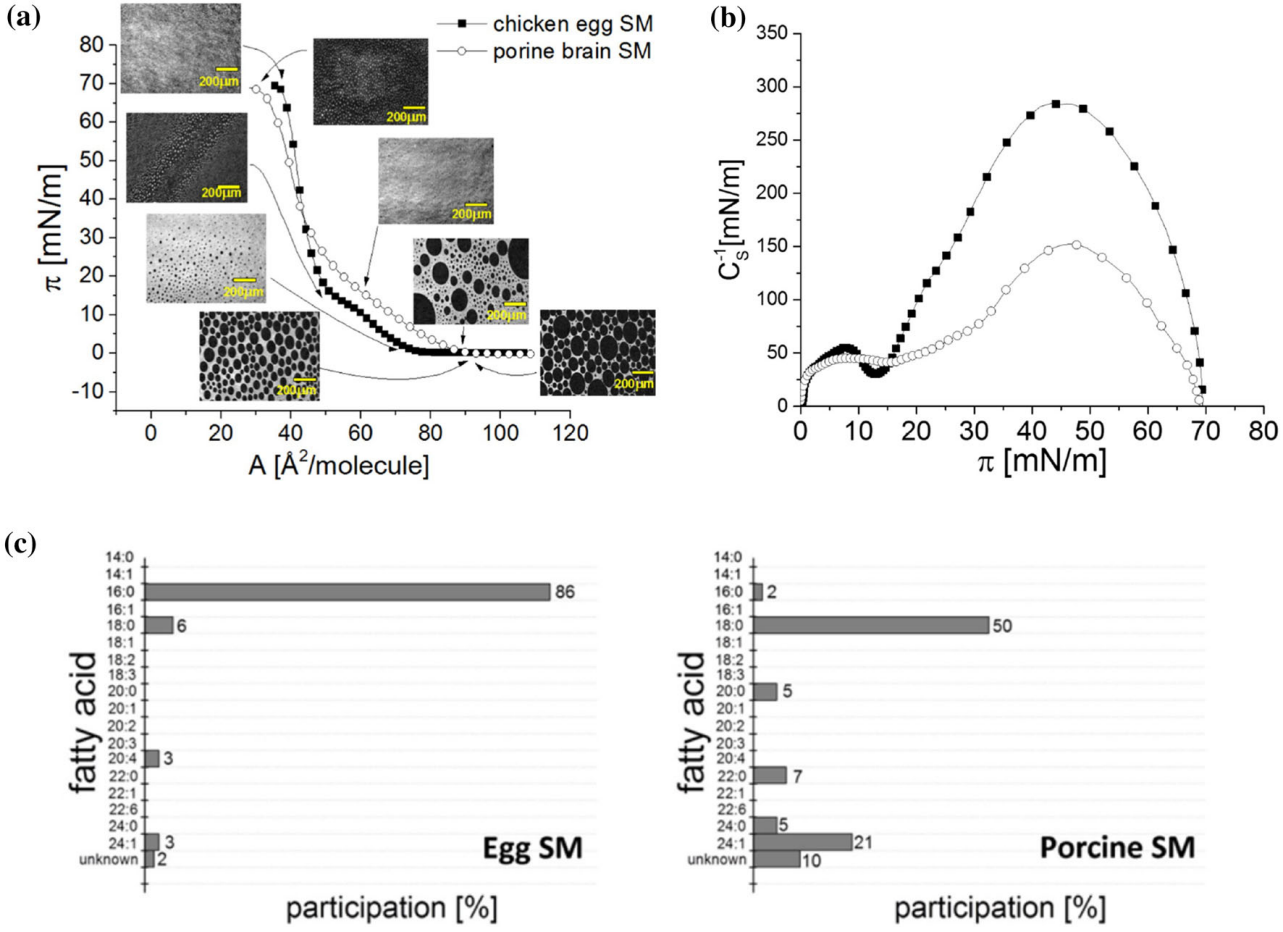
Surface pressure (π)–area (A) isotherms of sphingomyelin from different sources spread at the water/air interface, at 20 °C. *Inset* compression modulus (*C*_s_^−1^)–surface pressure (π) dependencies. *Below* comparison of fatty acid profiles of egg and porcine brain sphingomyelin

Figure 2 compiles isotherms recorded for both SM sources. Although monolayers from both kinds of SM show similar film characteristics (the presence of liquid-type transition that spans over molecular areas from 90/80 Å^2^/molecule to 50 Å^2^/molecule and collapse occurring at 69 mN/m), the striking difference is their physical state above the phase transition. Namely, porcine brain SM is more expanded (attains liquid-condensed state of *C*_s_^−1^_max_ = 150 mN/m at the most), while egg SM is capable of forming solid monolayers, of *C*_s_^−1^_max_ = 300 mN/m at higher pressure region. The lift-off area is also different: 78 Å^2^ versus 91 Å^2^ for egg and pork brain SM, respectively. The observed differences in monolayer characteristics for both SM samples are not surprising as it has been proved that hydrophobic chain structure (length and degree of unsaturation) strongly influences the character of the π/A isotherm of sphingomyelin (Ramstedt and Slotte 1999; Li et al. 2000). The course of the isotherm recorded for natural sample (which is composed of the mixture of different acyl chain sphingomyelins) differs from that for a synthetic one with specific hydrophobic chains (Vaknin et al. 2001). From Fig. 1, it is evident that fatty acid distribution in egg SM is different as compared to that isolated from porcine brain, i.e., porcine brain SM contains more (of about 7%) unsaturated fatty acids as compared to egg SM. This causes the latter monolayer to be significantly more expanded.

Another membrane lipid investigated here, POPC, forms a liquid-expanded monolayer without any visible transition in the course of the isotherm and has a lower collapse pressure (50 mN/m) as compared to the saturated phospholipid (DPPC). The isotherms (incorporated in Fig. 4, next section) agree with those published in literature (e.g., Yun et al. 2003).

Cholesterol has been thoroughly characterized in Langmuir monolayers and the isotherms recorded herein agree very well with those published elsewhere (e.g., Cadena-Nava et al. 2006; Minones Conde et al. 2010). Since in the literature much less attention has been paid to 7-KC, we have thoroughly investigated this oxidized sterol in Langmuir monolayers.

Surface pressure/area (π/A) isotherms registered for 7-KC and Chol (for comparison) spread on the air/water interface at 20 °C, complemented with Brewster angle microscopy (BAM) images, are presented in Fig. 2a, b, which also contains the compression modulus (*C*_s_^−1^) versus surface pressure (π) dependencies (*C*_s_^−1^ is defined as $- A\frac{d\pi }{dA}$ (Davies and Rideal 1963)).

**Fig. 3:**
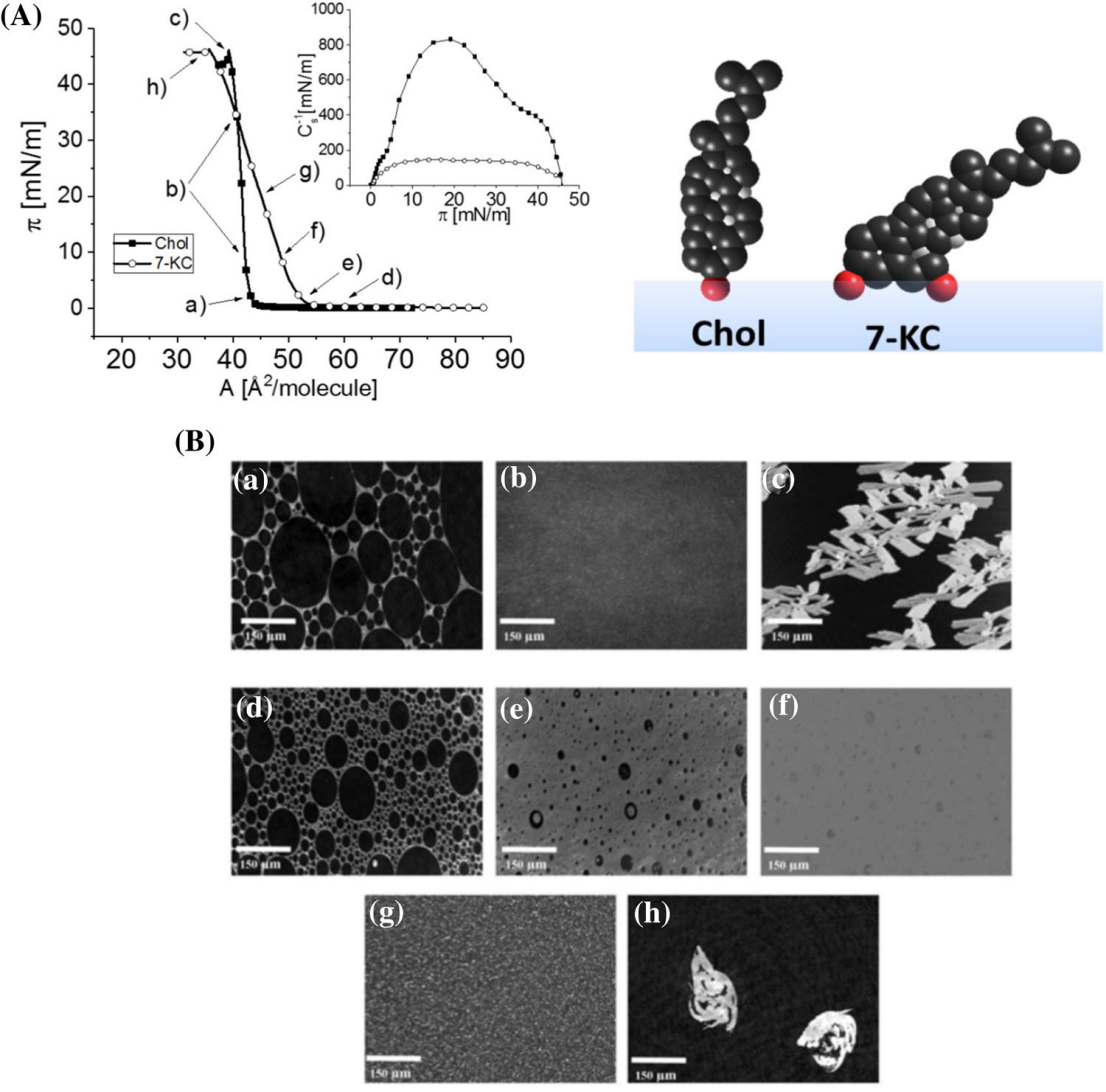
**A** *Left*—surface pressure (π)–area (A) isotherms of cholesterol (Chol) and 7-ketocholesterol (7-KC) spread at the water/air interface, at 20 °C. *Inset* compression modulus (*C*_s_^−1^)–surface pressure (π) dependencies;* right*—possible orientation of the molecules at the air/water interface. **B** BAM images recorded at the selected moments of the monolayer compression for Chol (*a*–*c*) and 7-KC (*d*–*h*)

The π–A isotherm registered for a 7-ketocholesterol spread on the free water surface at 20 °C is in good agreement with the data published by Mintzer et al. (2010). Although 7-KC is structurally very similar to cholesterol, and the only difference is the presence of carbonyl group in the ring at the seven position, the isotherm characteristics for both compounds are significantly different. Firstly, π–A isotherms differ in the value of the ‘lift-off’ point (area at which the increase of surface pressure above zero is observed) as well as in the value of limiting area (determined by extrapolation of the last rectilinear segment of isotherms to the surface pressure which equals zero). Secondly, sound differences are also seen in films’ compressibility, i.e., maximum values of compressibility modulus reach 830 mN/m and 160 mN/m for cholesterol and 7-KC, respectively. This means that cholesterol monolayer is significantly more condensed as compared to its oxidized derivative. This is quite understandable taking into consideration different possible orientations of the investigated sterol molecules at the air/water interface (Fig. 3A, right panel). Orientation of cholesterol in Langmuir monolayers is well known—molecules are oriented vertically at the free water surface. However, the presence of an additional polar group is supposed to change the alignment of molecules. From molecular models, it seems that molecules of oxidized sterols are more tilted with respect to the surface. Due to a larger area of polar part, their limiting area is increased (which indeed is observed in the pressure/are isotherms). Different molecular tilt and bulkier polar part cause the monolayers from 7-KC to expanded more as compared to cholesterol. Less condensed monolayer formed by 7-KC molecules is also confirmed by BAM images. Both sterols show gas–liquid coexistence at the very low surface pressure region (below 1 mN/m; images a and d in Fig. 3B) and, upon compression, film of cholesterol immediately transforms into solid monolayer (b), which further collapses (c). However, the monolayer composed of 7-KC first undergoes a transition to liquid-expanded state (e) and then to liquid-condensed state (f). At surface pressures of about 20 mN/m, nucleation prior to collapse (h) is observed. One common parameter of investigated monolayers is their collapse pressure, which occurs at around 46 mN/m.

We have also performed a number of systematic studies showing the influence of experimental conditions on the characteristic of the π/A isotherm from 7-KC and the obtained results are shown in Fig. S1a–c.

It has been observed that changing the number of molecules deposited on the surface does not influence the shape of π–A curves (Fig. S1a). The *C*_s_^−1^ versus π dependence shows that the monolayer attains a liquid-condensed state. Afterwards, we have studied the effect of a barrier speed on monolayer characteristics (Fig. S1b). It has been found that the variation in compression rate (within the range of 15–30 mm/min) slightly influences the region of higher surface pressures (above 25 mN/m). However, the decrease of the compression speed to 10 mm/min induces monolayer nucleation at low pressures (around 20 mN/m). The influence of subphase temperature has also been examined. As expected (Fig. S1c), subphase temperature affects the collapse pressure value, which is the lowest (43 mN/m) at 30 ºC, while the highest (47 mN/m) at 10 °C. Finally, the influence of subphase pH was investigated. Our experiments (not shown here) indicate that a change of subphase pH within the range of 3–7 has no influence on the monolayer characteristics. Results of the above experiments clearly indicate that 7-KC is a good film-forming material, suitable for investigations with the Langmuir monolayer technique.

### Characteristics of 2-Component Lipid Mixtures

#### POPC and SM Mixed with Cholesterol versus 7-KC

**Fig. 4:**
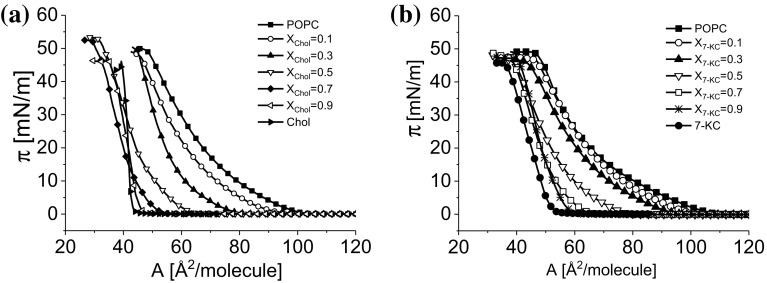
Surface pressure (π)–area (A) isotherms for Chol/POPC (**a**) and 7-KC/POPC (**b**) mixed monolayers

**Fig. 5:**
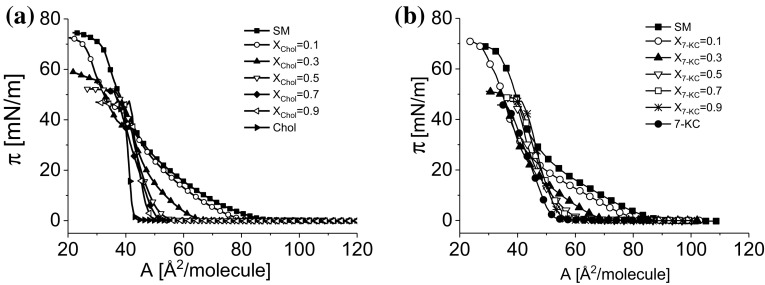
Surface pressure (π)–area (A) isotherms and the collapse pressure (π_coll_) versus mixed film composition plots for brain SM with Chol (**a**) and brain SM with 7-KC (**b**)

The results of Langmuir monolayer experiments for Chol/POPC, 7-KC/POPC, Chol/SM, and 7-KC/SM are presented in Figs. 4 and 5.

Isotherms presented in Figs. 4 and 5 show that both Chol and 7-KC exert a condensing effect on both SM and POPC monolayers. The contour of isotherm changes systematically upon the addition of 7-KC, from shape corresponding to pure POPC (or SM) to that characteristic for the investigated oxysterol. In all studied systems, collapse pressures change with film composition, indicating miscibility between film components.

To quantify the interactions, the values of the excess free enthalpy changes (∆G^*exc*^) were calculated using the following formula (Pagano and Gershweld 1972):

$$ \Delta G^{Exc} \; = N{}_{A}\;\int\limits_{0}^{\pi } {A^{Exc} d\pi }, $$

where $$ A^{Exc} = A_{12} \; - \;(A_{1} X_{1} + A_{2} X_{2} ). $$

Here X_1_ (X_2_) is the mole fraction of component 1 (component 2), A_12_ means the molecular area in mixed film, A_1_(A_2_) is the area occupied by molecule in one-component film, and N_A_ is the Avogadro number.

**Fig. 6:**
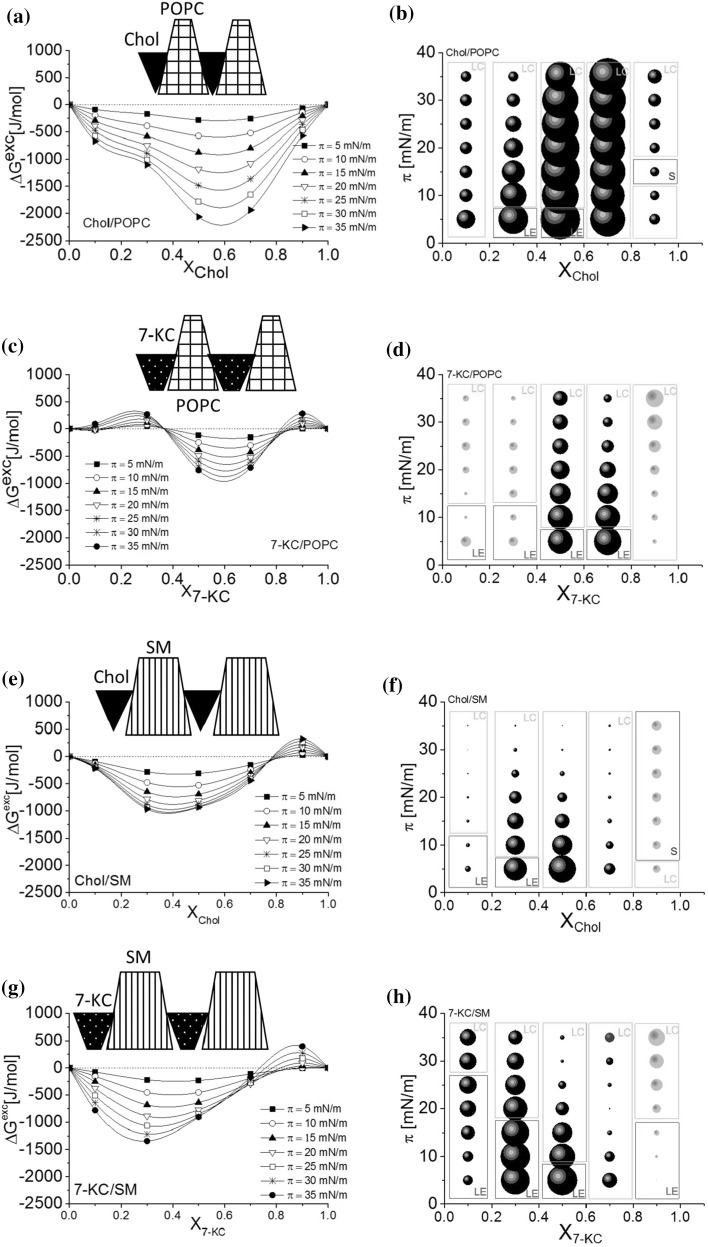
Excess free enthalpy of mixing (∆G^exc^) as a function of film composition for mixtures of Chol or 7-KC with POPC and SM (**a**, **c**, **e**, **g**) together with schematic representation of geometry of interacting molecules, and the effect of the surface pressure and composition on the interactions of Chol or 7-KC with SM and POPC determined at 20 °C (**b**, **d**, **f**, **h**). The diameter of the bubbles is proportional to the value of excess area per molecule (A^exc^). *Bright bubbles*—positive values, *dark bubbles*—negative values; the physical state of monolayers was determined on the basis of compression modulus values: *LE* liquid expanded, *LC* liquid condensed, *S* solid state

Interactions in each investigated system are illustrated in the ΔG^exc^ = f(X_sterol_) plots (left panel, Fig. 6a, c, e, g) and A^exc^ values (represented as bubbles, which area is proportional to the excess area per molecule value) are plotted as a function of surface pressure and film composition (right panel, Fig. 6b, d, f, h).

The obtained results clearly indicate that in mixtures of the investigated sterols with POPC the largest deviations from ideality (the largest A^exc^ values) occur in the region of a film composition (expressed as X_sterol_) ranging from 0.5 to 0.7, which corresponds to the strongest attractive interactions between POPC and sterol molecules (minimum ΔG^exc^ values). For POPC/Chol, ∆G^*exc*^ values are negative in the whole composition range, while in the case of mixtures with 7-KC negative values occur within the range 0.4–0.8. Although the minimum appears at the same film composition for POPC mixed with both sterols, the strength of interaction with Chol is nearly twice that for the 7-KC.

For mixtures with SM, the course of the ∆G^*exc*^ = f(X_sterol_) function is similar for cholesterol as well as its oxidized derivative, and the strength of interaction in both cases is also comparable, although slightly stronger attractive interactions are observed in 7-KC-containing mixtures. Negative values are observed for X_sterol_ < 0.7 with minimum at ~0.3, which corresponds to the 2:1 (SM: sterol) ratio of the components. It is known that sterols and sphingomyelin have high affinity to each other—cholesterol has been most frequently examined in this respect (Smaby et al. 1994; Petelska and Figaszewski 2013). Strong attractive interactions between both lipids have been evidenced; the strongest are observed for Chol–SM films in a 1:2 proportion (Hąc-Wydro and Dynarowicz-Łątka 2008). It has been postulated that at this particular composition highly stable “surface complexes” between cholesterol and sphingomyelin are formed. Therefore, this composition is attributed to the lipid raft model in mammals.

**Fig. 7:**
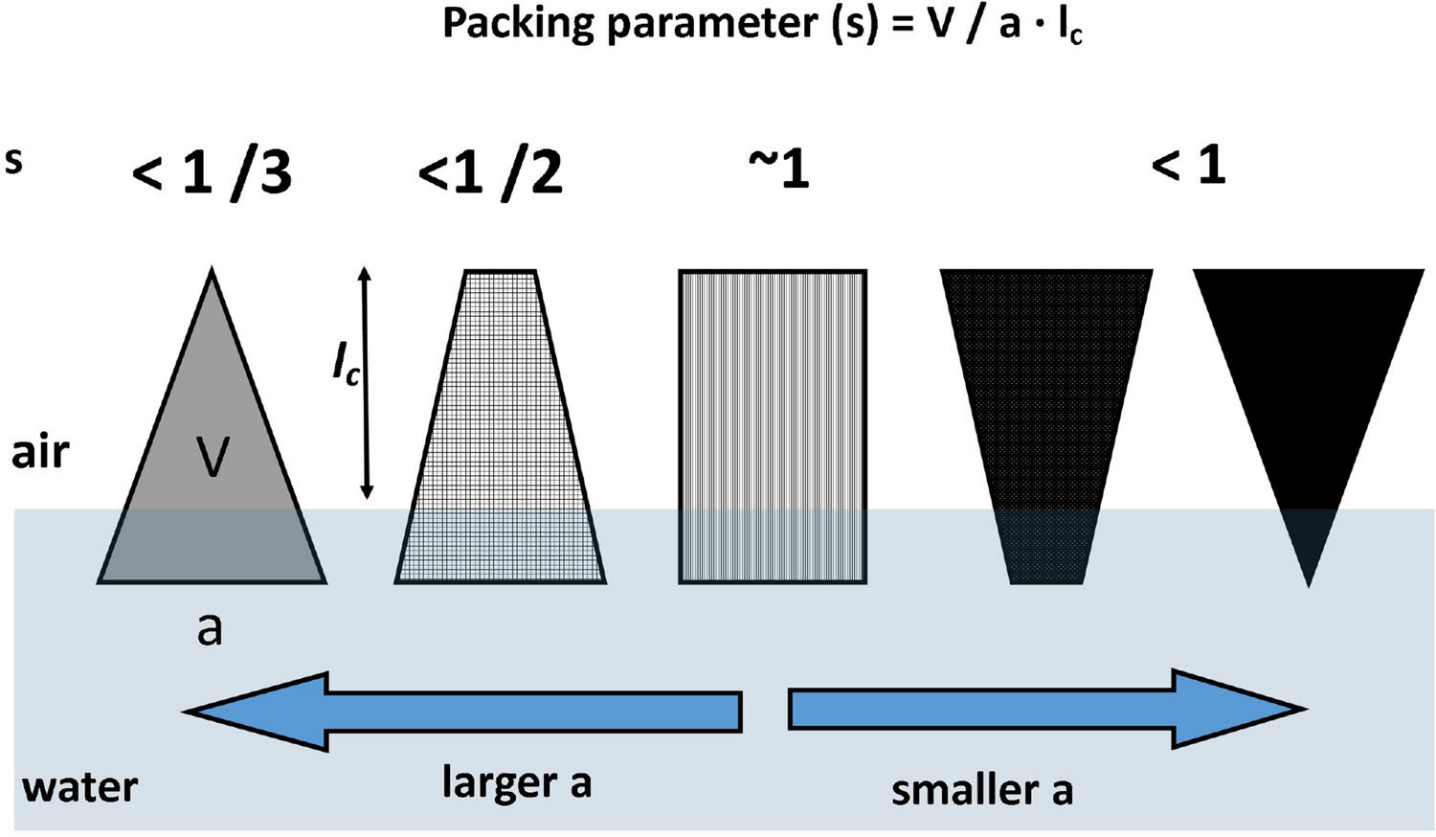
Mean packing shapes of molecules (according to Israelachvili 2011)

In order to explain the observed differences in interactions, we have analyzed geometric packing of molecules, which is expressed in terms of a dimensionless critical packing parameter *s* (defined as $s = \frac{V}{{a \cdot l_{c} }}$ (Israelachvili 2011) that depends on the head group area *a*, volume *V*, and critical length *l*_*c*_ of the hydrocarbon chain. From the value of *s*, it is possible to assign the shape of a molecule (Fig. 7).

Geometry of phosphatidylcholines (PCs) has been estimated to be conical (Israelachvili 2011). This ensures favorable packing with cholesterol, which is of opposite geometry (usually modeled as inverted cone (Israelachvili 2011)). In this system, the existence of strong intermolecular interactions is quite understandable. However, sphingomyelin has a truncated cone shape (Israelachvili 2011). Therefore, in mixtures with cholesterol, shape complementarity is slightly worse as compared to those with PC, which reflects in weaker interactions due to a looser mixed monolayer (differences in condensation of mixed monolayers Chol/POPC and Chol/SM can clearly be seen in Fig. 6b, f).

Despite the fact that thermodynamic analysis of interactions agrees well with geometry consideration, one has to bear in mind that molecular shapes are estimated from the values of critical packing parameter, which does not take into account specific interactions between mixture components, i.e., electrostatic forces, hydration, chain motion. However, such analysis based on geometric packing of molecules indicates a general trend of molecular arrangements in monolayers.

7-KC has a larger polar part area as compared to its non-oxidized form. Consequently, critical packing parameter will be smaller than that for cholesterol, and its shape will be different (inverted truncated cone). In mixtures with POPC or SM, shape complementarity will not be as optimal as for cholesterol. As a result, mixed monolayers are looser and the interactions are weaker than in cholesterol-containing systems.

### Characteristics of Ternary Sterol/POPC/SM Systems

Cholesterol, POPC, and SM are main lipids in neurons, and therefore such a ternary mixture may serve as a model of neuronal membrane. Our previous experiments (Dynarowicz-Łątka et al. 2015) proved that the 2-component system composed of POPC and SM is not stable from the thermodynamic point of view as it is characterized by weak repulsive interactions. This agrees well with the analysis of the geometry of interacting molecules as the arrangement of conically shaped PC with SM of a truncated cone shape cannot be considered the most favorable.

**Fig. 8:**
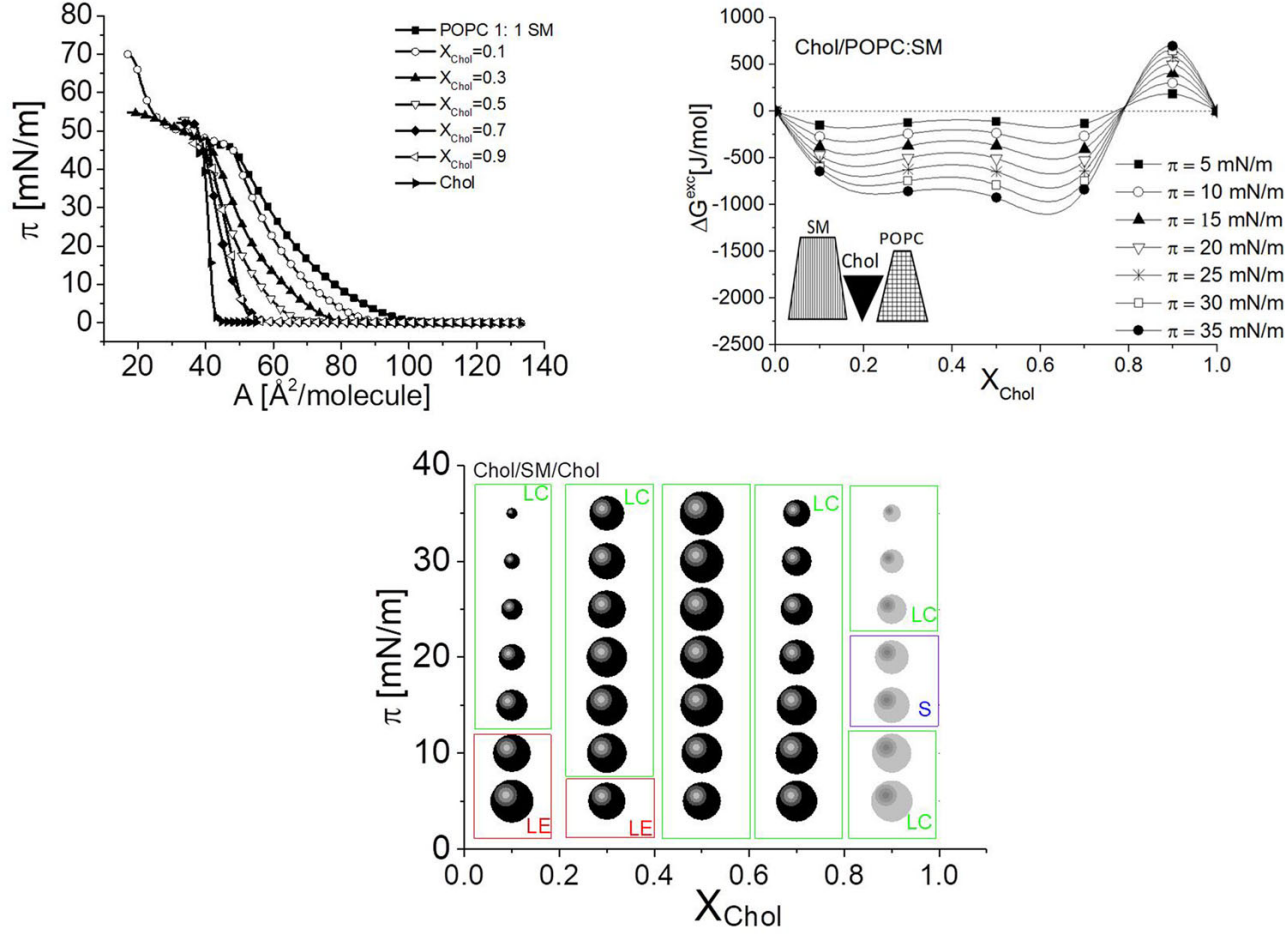
Surface pressure (π)–(A) isotherms and ∆G^exc^ versus mixed film composition (X_chol_) plots for the ternary system Chol/POPC/SM and the effect of surface pressure and composition of the mixed layers on the interactions between Chol, SM, and POPC

**Fig. 9:**
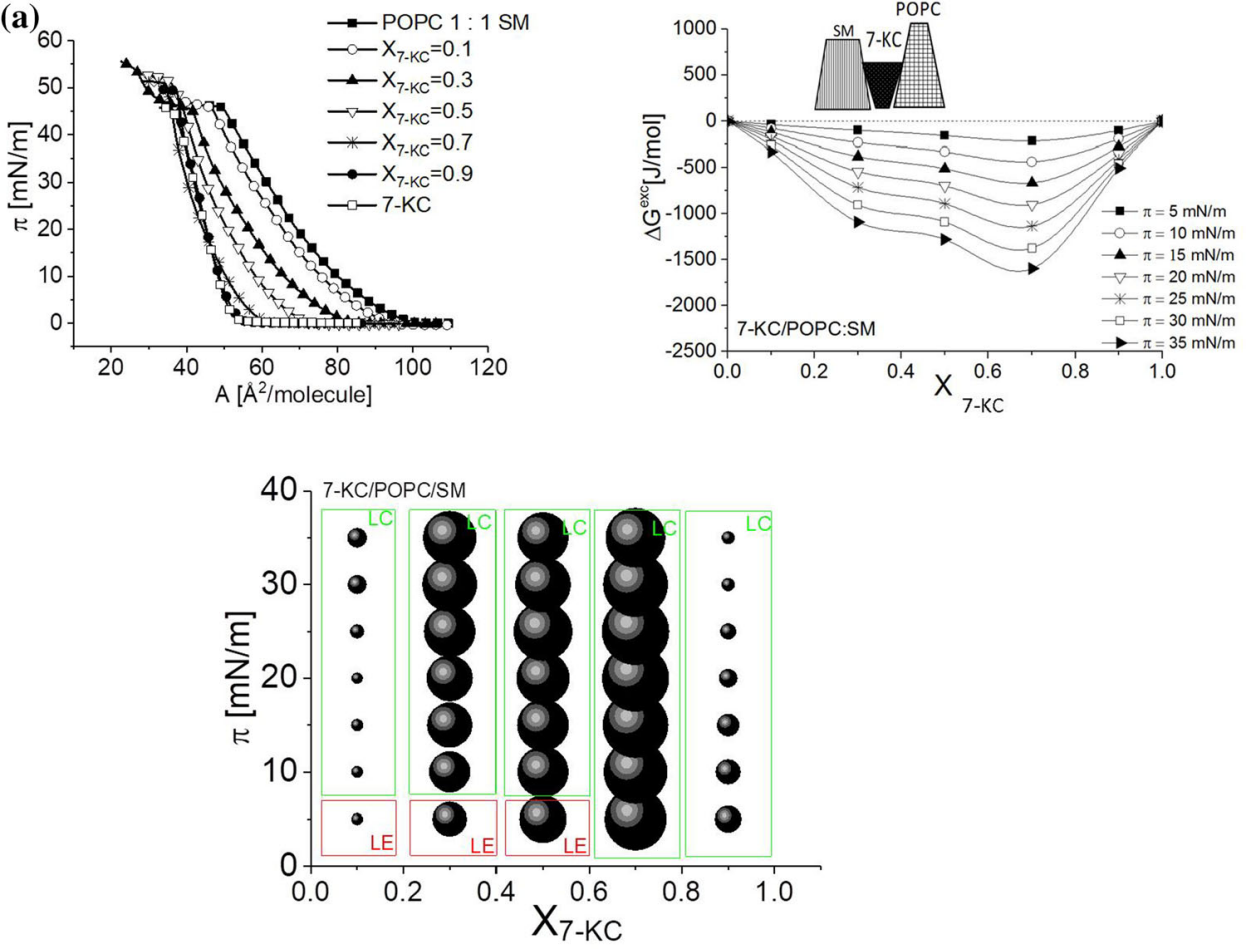
Surface pressure (π)–(A) isotherms and ∆G^exc^ versus mixed film composition (X_7-KC_) plots for the ternary system 7-KC/POPC/SM and the effect of surface pressure and composition of the mixed layers on the interactions between 7-KC, SM, and POPC

Experimental isotherms and the calculated ∆G^*exc*^ = f(X_Chol_) plots recorded for ternary system containing POPC and SM (mixed in 1:1 ratio) and cholesterol in different amounts are shown in Fig. 8. To compare the behavior of 7-KC versus cholesterol, we have constructed an analogical ternary mixture and the results are presented in Fig. 9.

It is evident that the introduction of sterol to the POPC:SM mixture stabilizes the system since, in both investigated cases (Chol/POPC/SM and 7-KC/POPC/SM), ΔG^exc^ values are negative in the whole range of sterol mole fraction, except for cholesterol-rich films of X_chol_ > 0.8. From geometry consideration, it is evident that the introduction of sterol molecule in-between POPC and SM improves the packing arrangement. Both ternary systems behave similarly in Langmuir monolayers. However, in the case of Chol-containing mixtures the interactions are significantly weaker as compared to those with 7-KC and seem to be independent of the ratio of cholesterol in a wide range of mixed film compositions.

Thus, it is clear that replacing cholesterol with 7-KC will enhance the interaction between molecules in the model membrane. This finding may provide some insight into understanding the process of neurodegeneration. It is known that optimal membrane fluidity is of utmost importance for normal cell functioning and any deviation (either increase or decrease) may result in a pathological state. There is a plethora of reports that relate changes in membrane fluidity to various diseases (Clarke et al. 1999; Hąc-Wydro and Dynarowicz-Łątka 2010a, b; Tekpli et al. 2013; and references therein). The stronger intermolecular interactions observed herein caused by incorporation of 7-KC can influence the physical properties of neuronal membrane, thus altering synaptic transmission, receptor binding, and other processes that contribute to neural dysfunction.

## Electronic Supplementary Material

Below is the link to the electronic supplementary material.

Supplementary material 1 (PDF 461 kb)
