# Supplementary material for: Oxysterols Versus Cholesterol in Model Neuronal Membrane. I. The Case of 7-Ketocholesterol. The Langmuir Monolayer Study
Source: J Membr Biol. 2017 Aug 31;250(5):553–64. doi: 10.1007/s00232-017-9984-8 (PMC5613072; doi:10.1007/s00232-017-9984-8)
Supplement: Supplementary file 1 — Supplementary material 1 (PDF 461 kb) [file 232_2017_9984_MOESM1_ESM.pdf]

# Supplementary Material 1

The Journal of Membrane Biology

Oxysterols *versus* cholesterol in model neuronal membrane. I. The case of 7-ketocholesterol. The Langmuir monolayer study

Anita Wnętrzak, Katarzyna Makyła-Juzak, Anna Filiczkowska, Waldemar Kulig, Patrycja Dynarowicz-Łątka *

*Corresponding author: ucdynaro@cyf-kr.edu.pl

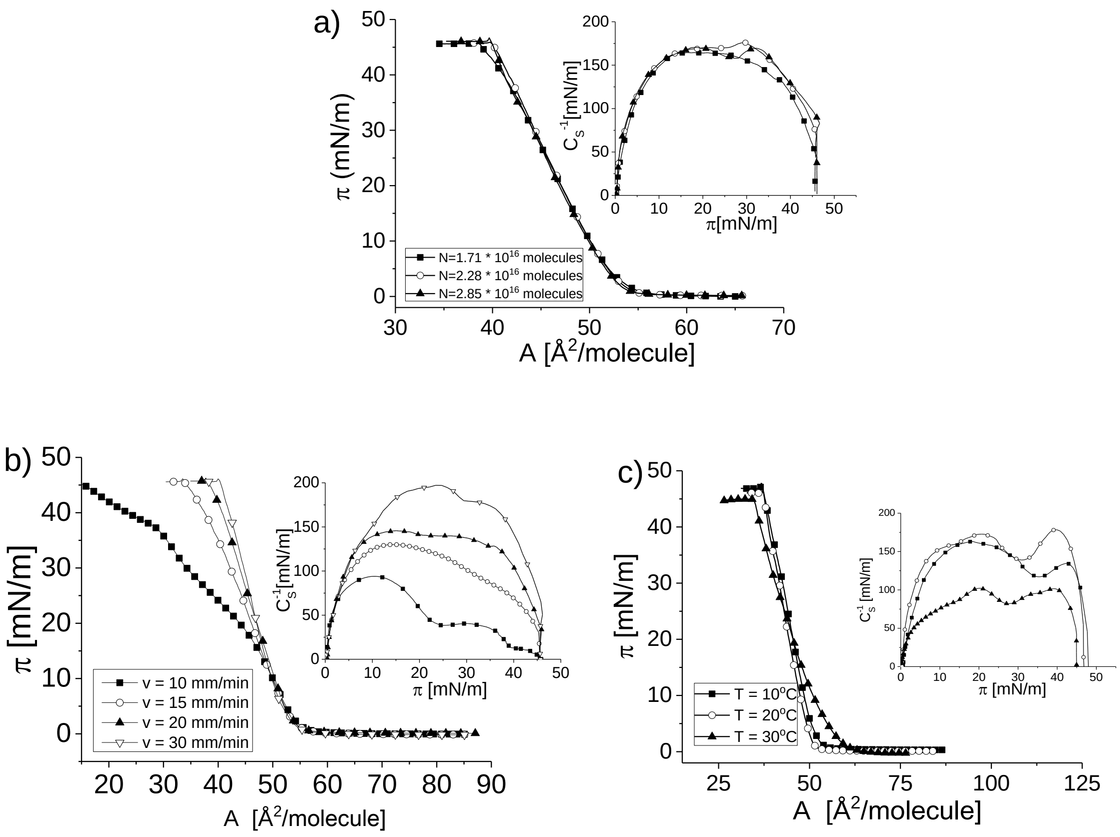


**Fig. S1** The influence of experimental conditions on the π-A isotherms of 7-KC: number of molecules (a), compression rate (b), temperature of subphase (c). Inset: compression modulus ($C_s^{-1}$) – surface pressure (π) dependencies.
